# Thoughts concerning the BMJ editorial "Kitemarking the west wind" and the WHO dot-health proposal

**DOI:** 10.2196/jmir.2.suppl2.e14

**Published:** 2000-12-12

**Authors:** Gunther Eysenbach

## Abstract

Tony Delamothe has recently written a BMJ editorial [1], which was partly inspired by the MedCERTAIN workshop in Heidelberg.

As the initiator and co-ordinator of an EU project, which could be misunderstood as a "kitemarking" project, I feel obliged to clarify what we want to achieve and how and why our approach differs from what is being described by Tony.

## An analogy to medical journals and their selection process

First some general remarks: it is interesting to note that every argument brought forward by Tony against the feasibility of selecting websites for recommendation to consumers can in principle be generalized to the problem of selecting, evaluating and endorsing *any* kind of printed or electronic information. If you replace "gateway/kitemark" in your article with "scholarly medical journal", you may find very similar arguments against the peer-review process. One could for example argue that to select medical manuscripts for publication in the BMJ is not only impossible because of the high volume of manuscripts (doesnít the BMJ receive 3000 manuscripts a year?), but also that it is a process that has similar problems of low inter-observer reliability (and none of the medical journals I am aware of have ever published figures regarding reliability and validity of their selection process or instruments used by reviewers). Despite these methodological difficulties, users (producers of the information as well as readers) appreciate the input of external evaluators and the input of an editorial board to improve the manuscripts. The scientific community has accepted peer-reviewed journals as a useful mechanism for users to access filtered information. As an aside, it is interesting to note that in the scholarly publishing world, the processes of evaluating the quality of the document (peer-reviewing) and making it physically available can also be reversed in the sense that papers may also be published first on pre-print servers and later be tagged with meta-information and post-publication reviews [2].

In a way, kitemarking, trustmarking and "gateway-building" on the web is a meta-publishing process, having a similar role to medical journals in selecting information that can be recommended to users. Such "selecting and publishing" processes will inherently always have a limited degree of "reliability". Traditionally, publishers have the task to take raw information, to establish quality control mechanisms and to establish a reputation for the reliability of their information. Gateways and trustmark concepts are meta-publishers who - without putting any ink to paper or producing another media - may get back to the fundamental truth of being a publisher: to be a credible source and to establish trust - in the future, "pure metainformation-publishers would evaluate and describe information produced by others on the Internet." [3]

This may illustrate that if a gateway, kitemark or trustmark enterprise is seen as a new form of publishing (with all of the subjective and unreliable decisions that are inherent in publishing scholarly material), rather than as a quality certification process, , we will have to live with the fact that we cannot achieve a process that has 100% reliability (in terms of 100% inter-observer agreement), much as journal editors and the scientific community live with this fact. Why should we expect a process intended primarily for consumers to be any more scientifically exact and reliable than the publishing process for scholarly material? This is not to say that I am not aware of the need for a questionnaire/instrument that has been tested for reliability and usability, which is in fact the first deliverable of the MedCERTAIN project.

## "You cannot evaluate, as the content changes..."

The argument that rating, evaluating, kitemarking websites is impossible because the content of websites changes too frequently is not a valid one, if the recommendation refers to the information system and information provider, but not the information itself. As an analogy to the argument that we may not recommend websites because the content changes too frequently one could also argue that it is impossible to recommend the BMJ or serials such as Clinical Evidence to colleagues, because the actual content changes frequently. However, I do recommend the BMJ to colleagues (without implying that I agree with everything that has been written in the journal!). The recommendation of such sources is based on knowing the process (and perhaps people) behind the information production and evaluating some samples of the product. If an evaluator has assessed both, he may decide to trust the information source, and may also communicate his opinion to others. Thus, the function of a trustmark would be "not to guarantee information correctness or usefulness, but to enhance trust in the information provider" [Heidelberg Consensus Recommendations on Trustmarks] [4]. Any trustmark approach can only indicate that the information producer has taken a number of steps to increase transparency and ensure quality, but cannot and does not intend to guarantee the "accuracy" of information.

## The MedCERTAIN approach is different: An analogy to food labels

Now, on what criteria can we select sites and information providers as being trustworthy? How this can be implemented can be best demonstrated by explaining the EU project MedCERTAIN (MedPICS Certification and Rating of Trustworthy and Assessed Health Information on the Net), which uses a "third-generation trustmark" concept. This must be discriminated from traditional "kitemarks" such as colourful logos awarded by the information provider himself in an act of non-monitored self-certification. We are well aware of the challenge of communicating this to users.

The MedCERTAIN labeling concept - a combination of self-labeling and third-party control - can be best explained by drawing an analogy to food labels. Replace for a moment the question of "which high-quality websites can we recommend to users?" with the question of "which high-quality food can we recommend to consumers?". Both questions seem to be unanswerable at first, as both to a large part depend on the needs of the individual concerned. It becomes obvious that we may not simply put labels on foodstuffs saying that, for example, this is "high-quality, recommended food". What is needed are elements of consumer education, self-labeling and external quality control.

**Figure 1:**
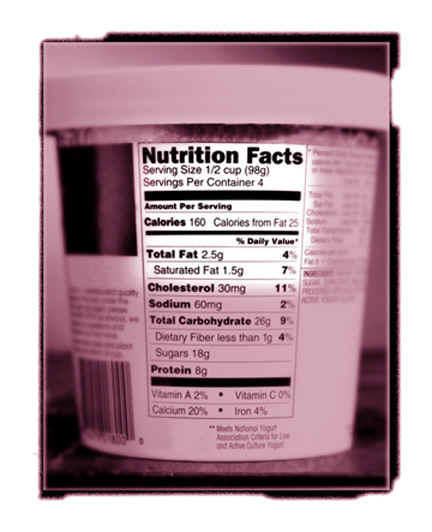
Food labels tell consumers what to expect inside the food package. In conjunction with consumer education and enforcement (making sure that labels are not abused as marketing tools) as well as third-party evaluations these labels help consumers to make informed decisions. (Image Source: FDA)

First, consumers have to be educated, to learn about the healthy components of a balanced diet. Different consumers may be interested in different things: for example, some may want to lose weight and may be taught that they should especially look for low fat products, others may suffer from hypertension and it may be stressed to them that they should avoid food rich in sodium.

Second, producers of food have to display the ingredients of their foods on standardized labels, telling consumers, for example, the amount of fat and sodium contained in the products.

Third, these labels and health claims of food providers have to a certain degree to be evaluated and compliance enforced. The US 1990 Nutrition Labeling and Education Act (NLEA), for example, regulates clearly what such nutrition labels should look like, when language such as "fat-free" may be used, which health claims may be used and how they have to be worded (http://vm.cfsan.fda.gov/label.html).

Only together, these measures empower and encourage consumers to make informed choices. The MedCERTAIN trustmark is hoped to play a similar role on the World-Wide-Web. The concept is also based on the pillars of consumer education, encouragement of self-labeling, and third-party evaluation [5,6]..

First, we have to educate consumers as to what they should look for. We may, for example, teach them about privacy issues or teach them about best practices in e-commerce, which internal quality management mechanisms in the production of information one may expect, and so on. The HON, AMA, IHC and Hi-Ethics group have all started to produce documents that may be used for these purposes, though their unclear language and lack of consensus has recently been critisized [7].

Second, information providers should display clear labels containing all the relevant information that allows consumers to assess the quality of an information provider. The first step in the MedCERTAIN trustmark project is therefore to let the information provider answer a questionnaire requiring them to disclose certain information, such as who is behind the information service, who are the significant sponsors, what are the internal quality processes, who is the target audience, what is the aim of the website, and so on. This meta-information will be presented to the consumer in a standardised and accessible format once he clicks on the MedCERTAIN trustmark, or may be retrieved from the browser in the background as meta-data. In analogue to nutrition labels, consumers may use the electronic MedCERTAIN label to select information that is relevant and appropriate for their individual needs and preferences. Moreover, MedCERTAIN will translate meta-information into computer-readable metadata, so that users may more specifically retrieve sites from search engines meeting their own needs, or to get alerts and advice if a site does not comply to individual preferences, similar to the W3C P3P process in the privacy field. This is the concept of "downstream-filtering" [8].

**Figure 2:**
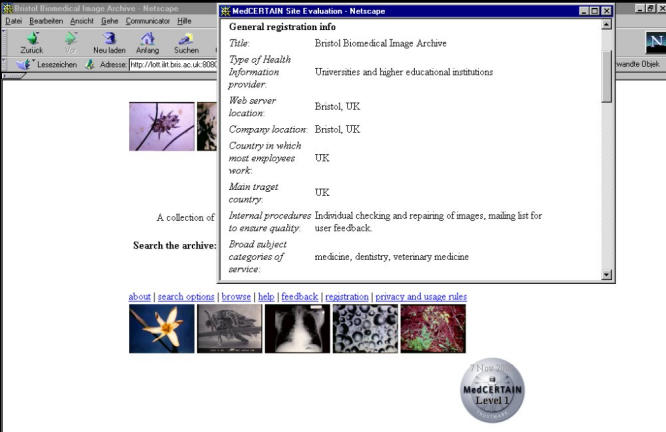
A click on the MedCERTAIN trustmark displays the MedCERTAIN label, which - similar to food labels - give users additional information about the site and the information provider. In addition, the same information will be distributed as computer readable, structured metadata (using the medPICS vocabulary)

On this level (MedCERTAIN level 1 trustmark), trust develops through self-commitment to web ethics and active disclosure in a standardized form and terminology. MedCERTAIN provides a stimulus and a technical framework for implementing web ethics, by providing a common access structure and terminology for disclosure information. Trust will be built up by the supplier of the information, as he tells users his processes on quality management, the qualification and training of staff, etc.

However, as with food labels, a certain degree of external control, monitoring and evaluation is necessary, otherwise anybody could abuse these labels for marketing purposes. It is a familiar phenomenon that many webmasters are abusing meta-data to increase traffic to their sites by using deceptive meta-information (e.g. by using the keyword "sex" in the description of their websites). Purely self-regulative initiatives such as HONCode are also sometimes abused as marketing instruments by webmasters, who may display the HON logo like an award, implying a degree of external evaluation that is not present. Pure self-certification systems may lead to more harm than good. Therefore, MedCERTAIN will, as much as possible, make sure that the meta-information provided by the information provider is correct, adequate and complete, and that formal ethical requirements of the site are complied with (level 2 evaluation).

Finally, experts will look at the actual product, i.e. evaluate the content of information sources (in what we call a level 3 evaluation), much as a gourmet tester may taste and review food for a consumer magazine. This is certainly the most difficult and disputed level of trust. The discussions at the Heidelberg workshop showed very clearly that quality criteria such as "accuracy" are difficult to operationalise - Tony is right in saying that these aspects of quality are difficult or impossible to be rated in a "reliable" and objective fashion. On the other hand, we think that it is irresponsible to imply endorsement of a website purely on grounds of structure and process, without making efforts to communicate to users what other people (preferably experts) say about a sample of the content. In cases of disagreement it is important for the consumer to be able to see which organisation or individual experts says what about a given resource. When the user clicks on a MedCERTAIN level 3 trustmark, he will therefore be able to access the meta-information on which organisation says what about a given site. While this is difficult to achieve in the real world (if I buy a book it takes considerable effort to find out which reviewer says what or which organisations recommend it), this is possible on the world-wide-web, if people who evaluate other resources use a common vocabulary (e.g. one expressed in XML). This vocabulary is the MedPICS vocabulary being developed as one deliverable of the MedCERTAIN trustmark. The idea is to build a "web of trust".

**Figure 3:**
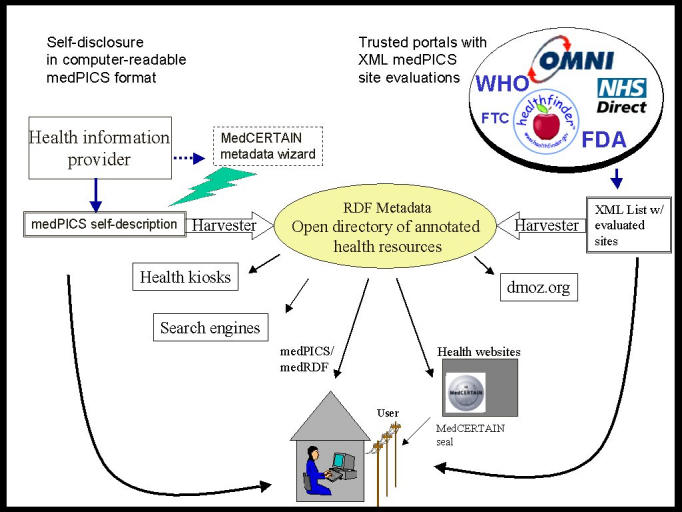
A common vocabulary to describe websites and health information providers such as medPICS enables users to view on the click of a button which organisation says what about a given site

## Do we really cope without infomediaries outside of the Internet?

The notion that we cope without gateways, kitemarks and other infomediaries outside the internet is wrong. People are recommending information, products and services to other people all the time. TV programmes, book reviews, seals of approval (e.g. from standardisation organisations), etc. are only some examples. In this context a question to Tony regarding the possible legal threat of people suing a gateway, kitemark or trustmark authority for not including their site on the gateway, or of consumers suing the gateway because they have been harmed by information providers endorsed by the gateway: How many journal editors do you know who have been sued over the "false-negative" rejection of an "acceptable" medical manuscript? And how many consumers have sued medical journals or referees because of "false positive" acceptance of a flawed article? I don't know of any case where this has been successfully tried.

The existence of the Web has led to an information overload for consumers, which has led to several authors arguing that health professionals should take the lead in guiding patients to the best available medical information on the web. This need has been recognized by governments such as in the USA (Healthfinder), UK (NHSDirect) and elsewhere to develop national gateways and other forms of infomediaries such as kitemarks or seals of approval. The universal challenge for developers of such systems is to establish a reliable and evidence-based process for selecting and recommending certain sites to consumers. A whole new scientific discipline is emerging around the question on how to appraise information for consumers, how to guide consumers to the best available information and how to help consumers in appraising information. We can either take a shortcut and lean back saying that appraising web information does not make sense and it is impossible to guide consumers to the best available information, or we can try to develop systems that guide consumers to the best information available, evaluate these systems and draw our conclusions.

**Figure 4:**
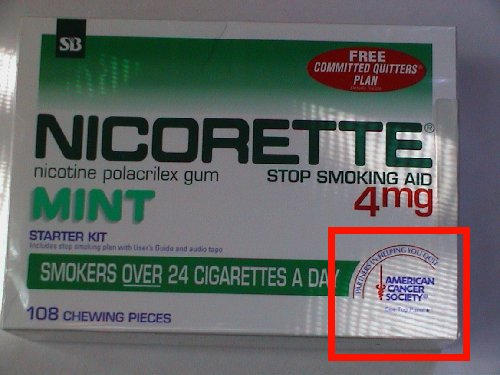
The notion that we cope without kitemarks and infomediaries outside the internet is wrong. People are recommending information, products and services to other people all the time. This is an example of a product endorsed by the American Cancer Society. The web allows to access and to display ratings, endorsements, evaluations of other organisations and individuals on the click of a button

**Figure 5:**
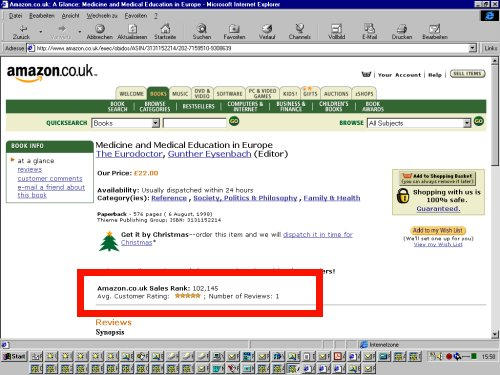
Book reviews (such as at Amazon) or TV-Guides are other examples for ratings, endorsements and evaluations of information in the real world

## The MedCERTAIN vs. the "dot-health" approach: Downstream selection versus upstream filtering

The MedCERTAIN trustmark concept, encouraging and monitoring the use of metadata to build a semantic web, is a decentralised, distributed system, allowing consumers to set their own preferences and needs and to match them against the information they find. This decentralised "rating" and endorsement of sites is a bottom-up (downstream filtering) quality process rather than a top-down (upstream filtering) process.

One example of a top-down, upstream filtering mechanism would be the central assignment of a .health top-level-domain (TLD) to health information providers approved by a central agency, as recently proposed by WHO [9]. Not surprisingly, this proposal has failed over concerns that "no single organisation should be entrusted to have a monopoly (real or perceived) over third-party verification of health-related information." And the viability of post-registration enforcement mechanisms [10].

As one participant of the electronic list discussing the proposal on the ICANN site rightly noted:

"if WHO feels they have a useful role to play by vetting health sites, then they should do so. Put on their web site a list of endorsed or approved sites with links. Those people who want to filter through WHO can do so. They can even have a "WHO Seal of Approval" which can be displayed only by approved sites. The public can use the vetting skill of WHO if they want to."

Indeed, we feel that a more viable approach would be for WHO to publish a list of approved and medPICS-XML tagged sites. This meta-information could be harvested and fed into search engines and systems like MedCERTAIN to generate a dynamic quality seal, allowing consumers on the click of a button to see which organisations have endorsed that site.

## Who pays the bill?

Certainly,all of this will come at some cost.: the volume of websites is a problem mentioned by Tony. However, the BMJ receives and reviews several thousands of manuscripts per year by using an international network of evaluators who work for free. The Cochrane Collaboration assesses and reviews thousands of clinical trials, also based on an international collaboration of volunteers. A similar approach may work on the web. The "Heidelberg Collaboration" is a recently established group intending to "help people, patients and professionals to identify health information useful to them" [11], e.g. by ensuring interoperability and common standards for selecting / recommending websites among portals / gateways and exploring possibilities for decentralised, distributed rating systems that take into account the power of the Internet.
